# Rule of UA on Cardiac Myocytes Uric Acid Differently Influence the Oxidative Damage Induced by Acute Exposure of High Level of Glucose in Chicken Cardiac Myocytes

**DOI:** 10.3389/fvets.2020.602419

**Published:** 2020-12-23

**Authors:** Xiaolong Sun, Hongchao Jiao, Jingpeng Zhao, Xiaojuan Wang, Hai Lin

**Affiliations:** ^1^College of Animal Science and Veterinary Medicine, Shandong Agricultural University, Tai'an, China; ^2^Shandong Key Lab of Animal Bioengineering and Disease Control and Prevention, Tai'an, China

**Keywords:** glucose, uric acid, antioxidant enzymes, cardiac cells, nuclear factor erythroid2-related factor

## Abstract

**Background:** Uric acid (UA) is a potent scavenger of oxidants in mammalian and avian species. In humans, hyperglycemia with simultaneous hyperuricemia may exert additional damage to the cardiovascular system. Chickens naturally have hyperglycemia (10.1–11.0 mmol/L) and hyperuricemia (100–900 μmol/L), which makes them an interesting model.

**Methods:** The aim of this study was to investigate the effects of UA on the oxidative damage induced by acute exposure of high level of glucose in chicken cardiac myocytes.

**Results:** Cell viability and the concentrations of thiobarbituric acid reactive substance (TBARS) were decreased by glucose treatment in a dose- and time-dependent manner. After acute exposure to high level of glucose (300 mM), a moderate level of UA (300 μM) increased cell viability and reduced TBARS and glutathione (GSH) content. Compared to the control or to independent high glucose (300 mM) or UA (1,200 μM) treatment, the concurrent treatment of high glucose and high UA significantly increased the TBARS, protein carbonyl contents, and ROS concentration, whereas it decreased the cell viability, superoxide dismutase (SOD) activity, and GSH content. In the presence of high glucose and UA, the nucleic protein expression of nuclear factor erythroid 2-related factor 2 (Nrf2) was decreased and the mRNA levels of the genes *cat, sod1, sod2, gss*, and *gclc* were downregulated.

**Conclusion:** In conclusion, acute exposure of high level of glucose induced oxidative damage in the cardiac myocytes of chicken. The present result suggests that an adequate level of uric acid is helpful in alleviating the acute oxidative damage that is induced by high glucose, whereas the inhibition of the Nrf2 pathway by a high level of uric acid may render the cardiac myocytes more vulnerable to suffering from oxidative damage.

## Background

Hyperglycemia promotes the accumulation of reactive oxygen species (**ROS**) through different pathways (1). The signaling pathways of diacylglycerol/protein kinase C/NADPH-oxidase are directly triggered by hyperglycemia and appear to play a pivotal role in diabetic complications due to the production of ROS, oxidative stress, and cellular death (2). Diabetes mellitus is associated with the increased risk of cardiovascular diseases, in which oxidative stress plays an important role (3, 4). The damage caused by ROS to cells is mainly characterized by the peroxidation of biological macromolecules, such as DNA (nuclear DNA and mitochondrial DNA), proteins (including enzymes), and lipid (especially unsaturated fatty) peroxidation (5, 6).

Uric acid (**UA**) is the end product of purine metabolism. In most species of mammals other than humans and primates, serum UA can be metabolized to allantoin by urate oxidase and then excreted from the kidney (7, 8). In contrast, urate oxidase is not present in avian species (9, 10) or in humans (11), which results in higher circulating UA concentrations in humans (200–400 μmol/L) and avian species (100–900 μmol/L) (12). Previous studies have confirmed that UA is an antioxidant agent in extracellular fluids (13–19). UA may also serve as a selective antioxidant both *in vitro* and *in vivo* to protect tissues and cells from oxidative damage (20, 21). UA is a potent scavenger of hydroxyl radicals and hypochlorous acid (21, 22). Mechanically, UA with normal concentrations can protect cells from oxidative stress by maintaining the enzymatic activity of catalase (CAT) and superoxide dismutase (**SOD**), which are important cellular antioxidant enzymes (23). However, UA has a “dual role” dependent on its concentration. For example, a physiological UA concentration partially alleviates oxidative stress in chicken cardiac muscle cells treated with H_2_O_2_, but abnormal elevated UA concentrations promotes oxidative damage in chicken cardiac muscle cells and aggravates oxidative stress in H_2_O_2_-damaged cells (24). An abnormal elevated concentration of UA can accelerate chain reactions of oxygen radicals and aggravate oxidative stress (25), and series of epidemiological investigations have proven a relationship between supraphysiological UA concentrations and angiocardiopathy, chronic kidney injury, heart disease, and metabolic syndrome diseases (26).

Hyperuricemia is an important clinical symptom that is thought to be related to cardiovascular disease and occur secondary to a state of generalized vascular endothelial dysfunction, and it has been shown to play a mechanistic role in cardiovascular disease by causing the inflammation and generation of oxidative stress in the vasculature (27). A growing evidence suggests that high circulating UA plays a causal role in the development of metabolic syndromes such as hyperglycemia, hypertriglyceridemia, and hypertension (28). In Japanese men, there is a positive correlation between serum UA and malondialdehyde, as measured by thiobarbituric acid-reacting substance (TBARS), a marker of systemic ROS production (29), suggesting a linkage of hyperuricemia and systemic oxidative stress.

In Wistar rats fed a high-fructose-enriched diet, the markedly reduced expression of catalase in the heart is accompanied with increased plasma UA (30), suggesting a possible link between insulin resistance and hyperuricemia. In hyperuricemia men, serum UA was an explanatory variable for serum glycated albumin (31). In women with type-1 diabetes, most oxidative stress metabolites are significantly increased, while plasmatic and urinary UA levels are significantly lower, and there is a significant negative relationship between Hemoglobin A1c (HbA1c) and plasmatic UA (32). UA can restore the hyperglycemia-induced decreased expression of G protein (the α-subunit of inhibitory guanine nucleotide regulatory protein) to control levels by inhibiting the production of NO and ONOO^−^, which may have benefit for the improvement of cardiovascular complications of diabetes (33).

Sturkie reported that the chicken emerged from the shell with a blood glucose level of 160–180 mg/100 mL (8.9–10.0 mM), which then continued to rise and reached an adult level of 180–240 mg/100 ml (10.1–11.0 mM) by the age of 2 months (34). Birds sustain levels of blood glucose 2–4 times higher than do mammals (35). So the chicken is a model of natural hyperglycemia and hyperuricemia. Although avian species have high metabolic rates, body temperatures, and blood glucose levels, they display longer lifespans than mammals with similar size (36). Avian cells demonstrated an enhanced resistance to 95% oxygen, hydrogen peroxide, and gamma-radiation when compared with mammals (37), and high circulating UA may afford birds against ROS-mediated damage (38). The increased gene expression of inflammatory cytokines is observed in chickens as a consequence of a reduction in plasma UA (39). Hence, we hypothesized that UA may interact with glucose, playing a role in the oxidative damage of cardiac cells.

Heart development is nearly complete in the late development stage of chicken embryo, and cultured embryonic cardiomyocytes can exhibit signaling responses similar to mature cardiomyocytes, which makes it possible to establish the research model *in vitro* (40, 41). In order to confirm whether physiological concentration of UA could relieve the oxidative damage in chicken cardiac muscle cells, cardiomyocytes prepared from 14-day-old chicken embryos were cultivated and treated with high concentration of glucose to establish an oxidative injury model firstly. Then, different concentrations of UA were added, and the effect on the oxidative damage in glucose-injured-cells was investigated to gain a comprehensively understanding of dual effects of UA.

## Materials and Methods

### Preparation and Identification of Chicken Cardiac Muscle Cells

Specific-pathogen-free (SPF) chicken eggs were purchased from Jinan SAIS Poultry CO., Ltd and incubated in an air incubator at 38°C. Chicken cardiac muscle cells were prepared according to previous studies with small modification (42). Briefly, 14-day-old chicken embryos were obtained, and the embryo hearts were cut into pieces, and then digested with 0.1% trypsin containing 0.25% EDTA (43). To separate cardiac muscle cells from the prepared mixed cells, the mixed cells were seeded in 75 cm^2^ cell culture plates and incubated at 37°C in cell cultural medium containing 90% low-glucose Dulbecco's modified Eagle medium and 10% fetal bovine serum for 1.5 h. After incubation, fibroblast cells were adherent to the plate. Then, the suspended cardiac muscle cells were collected by centrifuge and distributed into 6-well cell culture plates and incubated in 5% CO_2_ at 37°C for 3 days prior to stress treatments.

To identify those chicken cardiac muscle cells, an indirect immunofluorescence assay (IFA) was performed. Briefly, prepared cells were washed with phosphate buffer solution (PBS) with three times, fixed with 4% paraformaldehyde and solubilized using 0.2% Triton X-100. Then, the cells were incubated with an anti-α-sarcomeric actin (Sigma, USA) monoclonal antibody (mAb) for 1 h at 37°C. At the same time, cells incubated with non-immune serum were used as control group. After washing with PBS with three times, the cells were incubated with a FITC-conjugated goat anti-rabbit secondary antibody for another 1 h at 37°C. Finally, cellular nucleus were stained with 4′,6-diamidino-2-phenylindole (DAPI) for 10 min and cells were observed using fluorescence microscope.

### Establishment of an Oxidative Damage Cardiac Muscle Cell Model and UA Treatment

To investigate the effect of UA on glucose-induced oxidative damage in chicken cardiac muscle cells, a glucose-damaged cardiac muscle cell model was established firstly. Cell culture medium were treated with glucose with concentrations of 5.5, 50, 100, 200, 300, and 400 mM for 6, 12, and 24 h, and the optimal concentration and time point were determined using a cell viability assay (CCK-8). Then, cells were, respectively, treated with 300 and 1,200 μM UA simultaneously with 300 mM glucose, which was the concentration determined by the aforementioned CCK-8 assay. The cultured cells were collected at 12 h after treatment to detect oxidative stress markers, activities of antioxidant enzymes, concentrations of antioxidant substances, and the expression levels of antioxidant enzyme-related genes.

### Cell Viability Assay (CCK-8)

To investigate the viability of cardiac muscle cells cultured in plates, the CCK-8 assay was performed according to instructions. Briefly, chicken cardiac muscle cells were cultured in 96-well-plates, and 100 μL of CCK-8 solution with a continuous 1/10 dilution was added to each well. Then, the plate was incubated in 5% CO_2_ at 37°C for 2.5 h. Absorbance in each well was measured at 450 nm by microplate reader (Elx808, BIO-TEK, USA). The mean optical density (OD) for each indicated group was used to calculate the cell viability percentage.

### Oxidative Stress Parameter Measurement

Contents of MDA and protein carbonyl, activities of antioxidant enzymes including catalase (CAT) and superoxide dismutase (SOD), and concentrations of antioxidants substances including glutathione (GSH) and ROS were selected as biomarkers of oxidative stress in this study. For MDA, a thiobarbituric acid reaction method according to previous studies with modifications was used (44). Briefly, cells were collected and suspended in sodium phosphate buffer and lysed by ultrasonication. Then, 8.1% sodium dodecyl sulfate, 20% acetic acid and 0.8% thiobarbituric acid (TBA) were added into reaction tubes, and the mixed liquids were incubated at 90–95°C for 1 h. After that, reaction tubes were cooled on ice, and a mixture of butanol:pyridine (15:1) was added. The absorbance was measured at 532 and 572 nm. The results were calculated, and expressed as μmol thiobarbituric acid reacting substance (TBARS) per g protein. For protein carbonyl, CAT, SOD and GSH, commercial kits purchased from Nanjing Jiancheng Bioengineering Institute (Nangjing, Jiangsu, China) were used to measure protein carbonyl and GSH contents and CAT and SOD activities according to the manufacturer's instructions. For ROS, UA or hydrogen peroxide treated cells were cultured in DMEM containing 10 μM 2′,7′-dichlorodihydrofluorescein diacetate (DCFH-DA) for 30 min in 5% CO_2_ at 37°C, and cells were observed using fluorescence microscope.

### ROS Concentration Detection by a Fluorescent Molecular Probe (DCFH-DA) Assay

A fluorescent molecular probe (DCFH-DA) assay was performed to detect the intracellular ROS concentrations. Cultured cells were treated with glucose or UA, and a fluorescent molecular probe (DCFH-DA) was added to cell culture medium for 30 min. Then, unincorporated probes in the medium were removed by washing with PBS, and cells were observed using fluorescence microscope. To quantitate intracellular ROS concentrations precisely, DCFH-DA-treated-cells were lysed with 700 μL of lysis buffer (Beyotime Biotechnology, Beijing, China) for 10 min, and protein concentrations were measured to calibrate the fluorescence signal by a BCA protein assay (Beyotime Biotechnology, Beijing, China). Then, cellular lysates were added into a 96-well-plate, and fluorescence signals were detected using a fluorescence microplate system (Perkin Elmer, Enspire 2300) with an excitation wavelength of 498 nm and an absorption wavelength of 522 nm. Relative fluorescence intensities were calculated using fluorescence intensity divided by protein concentration, and intracellular ROS concentrations were compared between different groups.

### Gene Expression Analysis by Quantitative Real-Time Reverse-Transcription Polymerase Chain Reaction (RT-PCR)

The mRNA expression levels of Nrf2 and antioxidant enzyme-related genes including catalase (CAT), superoxide dismutase 1 (SOD1), superoxide dismutase 2 (SOD2), glutathione peroxidase 1 (GPX1), glutathione peroxidase 7 (GPX7), glutathione synthetase (GSS), glutamate-cysteine ligase catalytic subunit (GCLC), glutamate-cysteine ligase modifier subunit (GCLM), and glutathione reductase (GSR) were determined by real-time PCR. The primers used in this study (Table 1) were synthesized by Shanghai Sangon Company (Shanghai, China) according to a previous study (24). Total RNA was extracted from cells and tissues using Trizol (Invitrogen Life Technologies, Carlsbad, CA). Then, 1 μg total RNA was reverse transcribed into cDNA using PrimeScript RT Master Mix (Takara, Dalian, China) following the manufacturer's instructions. The reaction system was prepared with a SYBR Green I master mix (Roche, Basel, Switzerland) and quantitative real-time RT-PCR was performed on an ABI 7500 Real-Time PCR System (Applied Biosystems, ABI, USA). The reaction parameters were listed as following: 95°C for 30 s, followed by 40 cycles of 95°C for 5 s and 60°C for 34 s. The specific products were confirmed by melting curves generated automatically using SDS analytic software (AVI). The relative expression levels of these genes were determined to the expression of chicken GAPDH mRNA using the 2^−ΔΔCT^ method.

**Table 1:** Gene-specific primers used for the analysis of my chicken cardiac myocytes gene expression.

**Gene**	**Sequences**	**Accession NO**.	**Product size (bp)**
CAT	F: GTTGGCGGTAGGAGTCTGGTCT	NM_001031215.1	182
	R: GTGGTCAAGGCATCTGGCTTCTG		
SOD1	F: TTGTCTGATGGAGATCATGGCTTC	NM_205064.1	98
	R: TGCTTGCCTTCAGGATTAAAGTGAG		
SOD2	F: CAGATAGCAGCCTGTGCAAATCA	NM_204211.1	86
	R: GCATGTTCCCATACATCGATTCC		
GPX1	F: TCACCATGTTCGAGAAGTGC	NM_001277853.1	124
	R: ATGTACTGCGGGTTGGTCAT		
GPX7	F: TTGCAATTACAGCACTCCTGCTC	NM_001163245.1	149
	R: TGCAACGTTGACAACTAACGACA		
GSS	F: GCTCAGTGCCAGTTCCAGTT	XM_425692.4	115
	R: GGTCCCACAGTAAAGCCAAG		
GCLC	F: CAACCACCCAACACTCTGG	XM_419910.3	130
	R: CTCTTGCCTCCTCTTCCTCA		
GCLM	F: CCTGAAGAAAGGGATGAACTG	NM_001007953.1	114
	R: CTGAGCAACTCCAAGGGAAG		
GSR	F: CCAGAACACCACCAGAAAGG	XM_001235016.3	114
	R: TTACCAAAGAGCCGAAGTGC		
Nrf2	F: TGACCCAGTCTTCATTTCTGC	NM_205117.1	186
	R: GGGCTCGTGATTGTGCTTAC		
GAPDH	F: CTACACACGGACACTTCAAG	NM_204305.1	244
	R: ACAAACATGGGGGCATCAG		

### Protein Extraction and Western-Blotting Analysis

The expression level of Nrf2 was determined by western blot analysis. Cells were collected and cytosolic and nuclear proteins were extracted using a commercial nuclear and cytoplasmic protein extraction kit (Beyotime Biotechnology, Beijing, China) according to the manufacturer's instruction. The proteins were denatured by heating in the water bath at 95°C for 5 min, and separated on 7.5% sodium dodecyl sulfate-polyacrylamide gel electrophoresis (SDS-PAGE) and transferred to a PVDF membrane (Millipore, USA). The membranes were blocked with 5% skim milk dissolved in PBST at room temperature for 1 h and incubated with anti-Nrf2 and anti-Lamin B1 mAbs (Abcam, Cambridge, MA) at 4°C overnight. The membranes were washed with PBST for three times and incubated with an HRP-conjugated secondary antibody (Sigma, USA) for another 1 h at room temperature. After washing with PBST three times again, positive reactions were detected by an enhanced chemiluminescence (ECL) detection system (Beyotime Biotechnology, Beijing, China). The band intensities were quantified using a VILBER Fusion FX5 Luminescence image analysis system (Vilber Lourmat, France) and compared with different groups.

### Statistical Analysis

All values were expressed as the means ± standard error of the means (SEMs). One-way analysis of variance (ANOVA) was performed using the SPSS statistical software package, version 17.0 (SPSS Inc., Chicago, USA). *P* < 0.05 was considered to be statistically significant.

## Results

### The Effect of UA on the Oxidative Damage Parameters in Acute Glucose Exposure-Damaged Cardiac Muscle Cells

**Figure 1:**
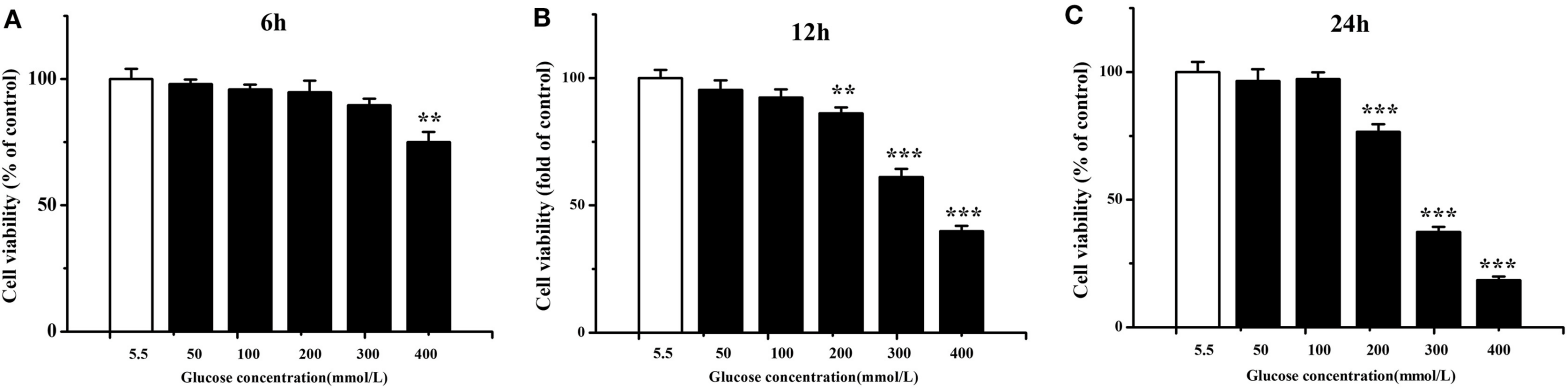
Viability of chicken cardiac muscle cells exposed to different concentrations (5.5, 50, 100, 200, 300, and 400 mmol/L) of glucose for 6 h **(A)**, 12 h **(B)**, and 24 h **(C)**. Data were expressed as the means ± SEM (*n* = 6); *P < 0.05, **P < 0.01, ***P < 0.001.

**Figure 2:**
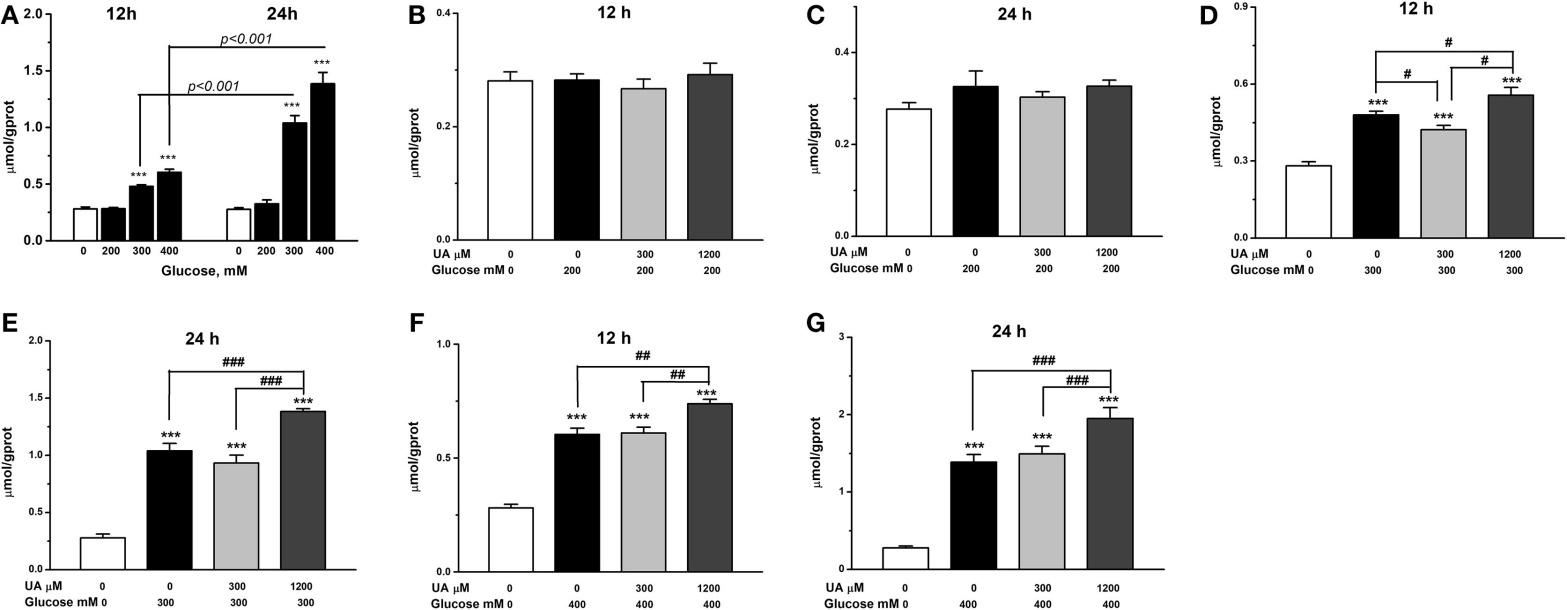
Effect of glucose alone treatments **(A)** and urate combined glucose treatments on lipid peroxidation of chicken cardiac muscle cells at 12 h **(B,D,F)** and 24 h **(C,E,G)**. Data were presented as the means ± SEM (*n* = 6). *P < 0.05; **P < 0.01; ***P < 0.001, compared with control not treated with urate and glucose; #*P* < 0.05; ##*P* < 0.01; ###P < 0.001, compared within glucose treatment groups. ^†*††*^*P* < 0.001.

**Figure 3:**
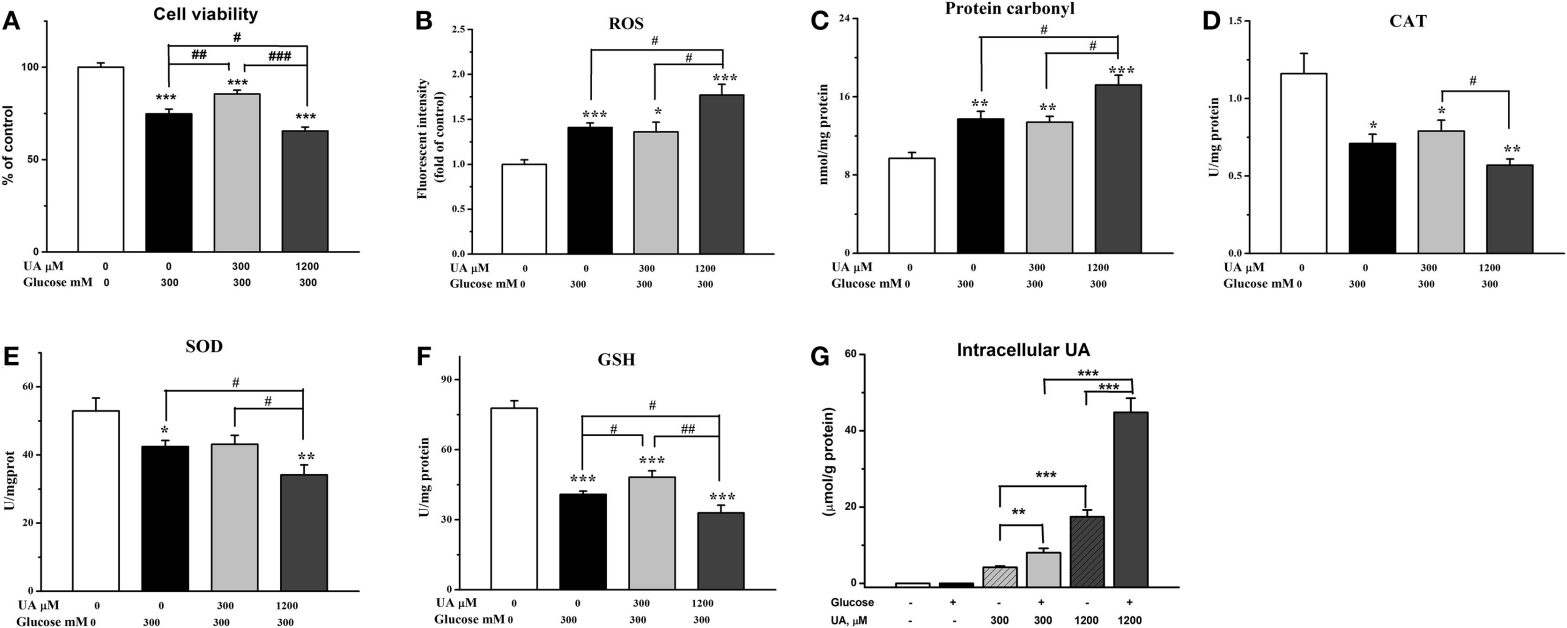
Effect of urate on acute exposure of high level of glucose- (300 mM) induced oxidative damage. **(A)** Cell viability, **(B)** ROS level (fluorescence intensity), **(C)** protein carbonyl, **(D)** catalase activity (CAT), **(E)** superoxide dismutase activity (SOD), **(F)** glutathione concentration (GSH), and **(G)** the intracellular concentrations of urate. Data were presented as the means ± SEM (*n* = 6). *P < 0.05, **P < 0.01, ***P < 0.001, compared with the control not treated with glucose and urate; #*P* < 0.05; ##*P* < 0.01; ###*P* < 0.001, compared within glucose treatment groups.

Chicken cardiac muscle cells were isolated from embryo heart tissues and identified by an IFA assay. To establish an oxidative damage cell model, cultured cardiac muscle cells were exposured to 5.5, 50, 100, 200, 300, and 400 mM glucose, respectively (Figure 1A). The results demonstrated that cell viability was decreased (*P* < 0.01) and the MDA concentration increased (*P* < 0.001) remarkably after 12 and 24 h of treatment with 200, 300, and 400 mM glucose (Figures 1B,C, 2A). MDA (Figure 2), protein carbonyl (Figure 3A) concentrations and ROS accumulation (Figure 3B) were significantly increased in cardiac muscle cells treated with 300 mM glucose for 12 h (*P* < 0.001), indicating the oxidative damage cell model was established successfully. In addition, in glucose-damaged cells treated with 300 μM urate for 12 h, TBARS significantly decreased (*P* < 0.05), while TBARS significantly increased (*P* < 0.05), when damaged cells were treated with 1,200 μM urate (Figure 2D). According to this result, 300 mM glucose was chosen as the working concentration in the subsequent experiments.

To investigate the effects of different concentrations of UA on the antioxidant function in oxidative damaged cardiac muscle cells, 300 or 1,200 μM UA combined with 300 mM glucose were added into cell culture medium. The results showed that the elevation in TBARS concentration by glucose was partially decreased (*P* < 0.05) (Figure 2D), and the decreased cell viability by glucose was increased by the addition of 300 μM UA (*P* < 0.01) (Figure 3A). In contrast, the cell viability was further decreased after UA treatment at a dose of 1,200 μM at the 12-h time point (*P* < 0.05) when compared with the glucose-challenged cells (Figure 3A). Correspondingly, the contents of both TBARS and protein carbonyl increased after the combined treatment of 300 mM glucose and 1,200 μM UA when compared with glucose-damaged cells (*P* < 0.05) (Figures 2D, 3C).

**Figure 4:**
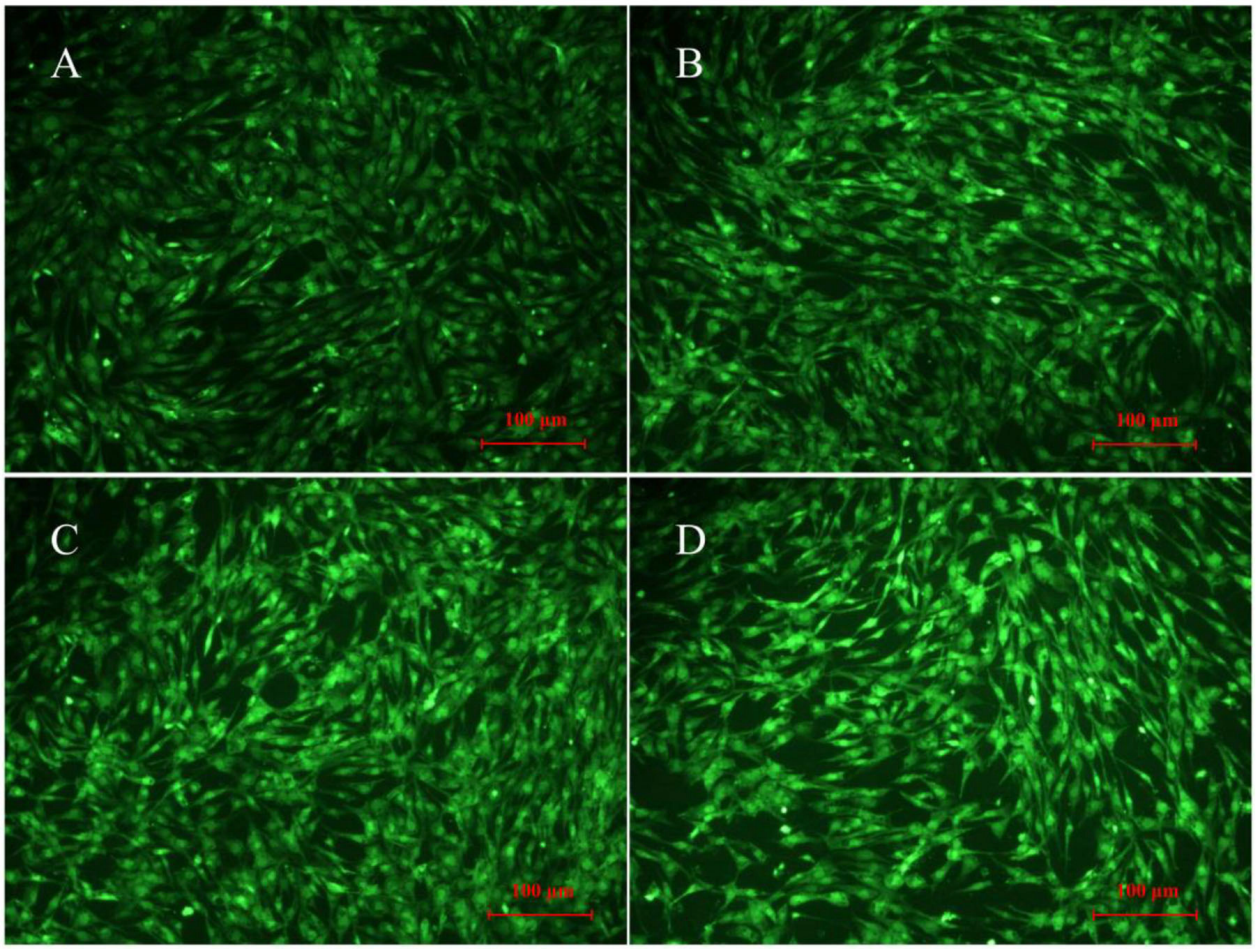
ROS production in normal chicken cardiac muscle cells (**A**, 400×), glucose-treated cells (**B**, 400×), glucose-treated cells treated with 300 μM UA (**C**, 400×), and glucose-treated cells treated with 1,200 μM UA (**D**, 400×).

After 12 h, the fluorescence signal was much stronger in glucose-treated cells than untreated cells (*P* < 0.001) (Figures 3B, 4A,B), indicating a higher accumulation of ROS in glucose-treated cells. There was no visible difference between cells treated with glucose combined with 300 μM UA and cells treated with glucose alone (Figures 3B, 4B,C). However, the fluorescence signal in cells treated with glucose combined with 1,200 μM UA was much stronger than that in cells treated with glucose alone (*P* < 0.05) (Figures 3B, 4B), and the cardiac muscle cells treated with 1,200 μM UA were changed from regular spindle-shaped cells into irregular strip-shaped cells (Figure 4D). These results demonstrated that a high concentration of UA aggravated intracellular ROS accumulation in chicken cardiac muscle cells.

### The Effect of UA on the Antioxidant System in Acute Glucose Exposure-Damaged Cardiac Muscle Cells

The results showed that, the GSH concentration was increased after 300 μM UA treatment for 12 h in glucose-damaged cells (*P* < 0.05) (Figure 3F), while there were no significant changes in CAT or SOD activities (Figures 3D,E). Correspondingly, both SOD activity and GSH concentration decreased 12 h after 1,200 μM UA treatment in glucose-damaged cells when compared with cells challenged with glucose alone (*P* < 0.05) (Figures 3E,F). CAT activity was not affected by 1,200 μM UA treatment in glucose-damaged cells (Figure 3D).

We also determined the concentrations of intracellular UA in control cardiac muscle cells and glucose-damaged cells. The intracellular UA level in 1,200 μM UA-treated cells was higher than that in 300 μM UA-treated cells (*P* < 0.001) (Figure 3G). Interestingly, the intracellular UA level in cells treated with 300 μM UA and glucose was much higher than that in cells treated with 300 μM UA alone (*P* < 0.01). Similarly, the intracellular UA level in cells treated with 1,200 μM UA and glucose was much higher than that in cells treated with 1,200 μM UA alone (*P* < 0.01) (Figure 3G), implying that glucose treatment increased the cell membrane permeability, making it easier for UA to enter into the cells.

### The Effect of Different Concentrations of UA on the Nrf2 Pathway in Acute Glucose Exposure-Damaged Cardiac Cells

**Figure 5:**
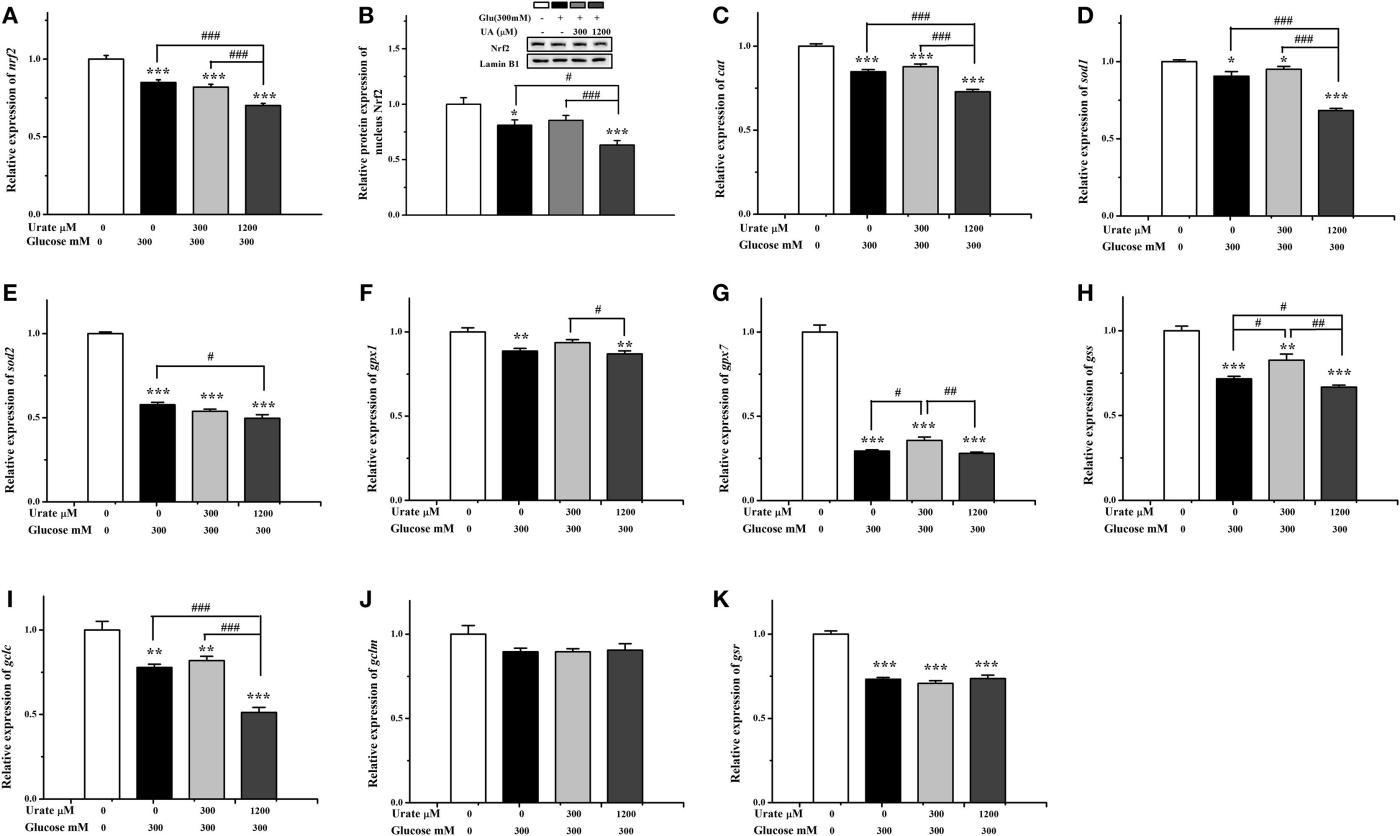
Effect of urate (0, 300, 1,200 μM) and high glucose (300 mM) treatment (12 h) on Nrf2 and downstream gene expression in chicken cardiac muscle cells. **(A)** The mRNA level of *Nrf2*, **(B)** the nucleus protein expression of Nrf2, **(C)** the mRNA level of *cat*, **(D)** the mRNA level of *sod1*, **(E)** the mRNA level of *sod2*, **(F)** the mRNA level of *gpx1*, **(G)** the mRNA level of *gpx7*, **(H)** the mRNA level of *gss*, **(I)** the mRNA level of *gclc*, **(J)** the mRNA level of *gclm*, and **(K)** the mRNA level of *gsr*. Data were presented as the means ± SEM (*n* = 6). *P < 0.05, **P < 0.01, ***P < 0.001, compared with the control not treated with glucose and urate; #*P* < 0.05; ##*P* < 0.01; ###*P* < 0.001, compared within glucose treatment groups.

The changes in the Nrf2/ARE pathway were analyzed (Figure 5), and the results showed that both mRNA (*P* < 0.001) and protein (*P* < 0.05) expression level of Nrf2 were decreased in glucose-damaged cardiac cells compared with the control cells (Figures 5A,B). In the presence of 300 μM UA, the glucose-suppressed expression levels of the Nrf2 protein showed no significant change compared with the control group (Figure 5B). Some genes downstream of the Nrf2/ARE pathway, including *gpx7* and *gss*, were partially increased compared with glucose-damaged cells (*P* < 0.05) (Figures 5G,H). In contrast, both the mRNA (*P* < 0.001) and protein (*P* < 0.05) expression levels of Nrf2 in glucose-damaged cells treated with 1,200 μM UA for 12 h were down-regulated compared with cells treated with glucose alone (Figures 5A,B), and the gene expressions of *cat* (*P* < 0.001), *sod1* (*P* < 0.001), *sod2* (*P* < 0.05), *gss* (*P* < 0.05), and *gclc* (*P* < 0.001) were also inhibited (Figures 5C–E,H,I).

## Discussion

In the present study, the antioxidative role of UA in chicken cardiac cells was evaluated. The results indicated that a normal physiological concentration of UA (less 300 μmol/L) was protective against acute exposure of high level glucose-induced oxidative damage. In contrast, a high concentration of UA (1,200 μmol/L) exacerbated acute high glucose exposure-induced oxidative damage by enhancing ROS formation and blocking the activation of the Nrf2 pathway. These results suggest that an adequate level of UA is a critical factor in protecting against oxidative damage to cardiac muscle cells and the pathological changes that result from this damage.

### Acute Exposure of High Level of Glucose Induces Oxidative Damage in Cardiac Muscle Cells

During the development of heart disease, oxidative stress is a major contributor in humans (45), rats (46), and chickens (47, 48). High blood glucose concentrations can induce oxidative stress, leading to chronic apoptosis and injuring tissue cells. High glucose levels can also independently increase the levels of 8-hydroxydeoxyguanosine (8-OHdG), an important marker of oxidative stress, in the plasma and urine of diabetic patients (49). In the present study, we established an acute model of glucose exposure (5.5–400 mmol/L, 6–24 h) and observed that the cell viability was decreased by glucose in a dose- and exposure time-dependent manner. The formation of lipid peroxidation reflected by thiobarbituric acid reacting substances (TBARS) was elevated by glucose treatment, suggesting that high glucose induces oxidative stress in cardiac myocytes. Similar results have been reported by previous works. In isolated adult rat ventricular myocytes that were incubated in a medium containing high concentrations of glucose (25 mM), the number of dead and apoptotic myocytes increased, whereas the content of glutathione decreased (46). But in difference, cell viability was decreased and lipid damage occurred until chicken cardiac muscle cells were treated with 300 mM glucose, suggesting the glucose tolerance in chicken cardiac muscle cells. The incubation of retinal Müller cells and bovine retinal endothelial cells in elevated glucose concentrations increases superoxide production, which is produced primarily by mitochondria (50). The ROS production and fraction of ROS-positive cardiomyocyte nuclei were drastically elevated in diabetic rats and normalized by antioxidant treatment (46). In the present study, the augmented ROS production, in concert with the suppressed activities of SOD and CAT and reduced antioxidant substances such as GSH, contributed to the enhanced oxidative stress caused by the high level of glucose treatment.

Several mechanisms have been proposed for the oxidative damage occurring during chronic hyperglycemia, including mitochondrial reactive oxygen species (ROS) overproduction (49) and the synthesis of advanced glycation end-products (51). Nrf2, a member of the cap “n” collar basic region leucine zipper (cnc bZip) group of transcription factors, is activated in response to a range of oxidative and electrophilic stimulants (52). After activation, Nrf2 translocates from the cytoplasm to the nucleus to bind to the antioxidant responsive element (ARE) sequences in relevant genes such as antioxidant genes, promoting their expression to enhance cell survival. In chickens, Nrf2 signaling is activated by H_2_O_2_ exposure (24) and involved in the maintenance of redox balance (53). The blocked Nrf2 signaling pathway in glucose treatment suggests that the deactivation of the Nrf2 pathway should be responsible for the impaired antioxidant capacity. Keap1-Nrf2-ARE signaling proteins play an important role in protecting cells from endogenous and exogenous stressors (54–56). The activation of Nrf2 and its downstream target genes protects the myocardium against oxidative stress induced by hyperglycemia (57) or hydrogen peroxide (24). Moreover, hyperglycemia enhances myocardial thioredoxin-interacting protein expression, possibly by reciprocally modulating p38 MAPK and Akt activation, leading to aggravated oxidative stress and the subsequent amplification of cardiac injury following myocardial ischemia/reperfusion (58). Recently, it has been proven that high-glucose-induced cardiac Nrf2 activation occurs through PKCα/PKCδ-ROS-JNK/p38 signaling (59). Hence, the signaling network associated with the oxidative stress that is induced by high levels of glucose needs to be investigated further.

### A Low Level of UA May Alleviate the Oxidative Damage Induced by Acute Exposure of High Level of Glucose

UA, as an end product of purine metabolism, acts as an antioxidant and accounts for 50% of the total antioxidant capacity of biological fluids in humans (60). In chickens, UA is an important contributor to the total antioxidant capacity in blood (61, 62), and it has been suggested to be one of the reasons for the increased longevity of birds relative to mammals of similar size (63).

In Wistar rats fed a high-fructose-enriched diet, the markedly reduced expression of catalase in the heart is accompanied with increased plasma UA (30). In clinical studies, circulating UA has been linked to serum glycated albumin in hyperuricemia men (31) and glycosylated hemoglobin in women with type-1 diabetes (32). Our previous study demonstrated that a physiological UA concentration partially alleviates the oxidative stress in chicken cardiac muscle cells treated with H_2_O_2_ (24). The present result further indicated that low levels of UA (300 μM) could decrease lipid peroxidation and partially restore the decreased cell viability and GSH concentrations by acute exposure of high levels of glucose, suggesting the beneficial effects of UA in the prevention of oxidative stress in cardiac myocytes. In a recent study, the protective effect of UA against oxidative damage in the central nervous system was investigated, and the results suggested that there is a negative association between UA levels and glaucoma severity in male patients (64). By comparing subjects with normal and high plasma UA, it has been suggested that circulating UA is a major antioxidant and may help protect against free-radical oxidative damage (65). An acute UA reduction caused a 25–40% increase in the levels of systemic and muscle-specific markers of oxidative stress (65). Inc women with type-1 diabetes, most oxidative stress metabolites are significantly increased while plasmatic and urinary UA levels are significantly decreased, and there is a significant negative relationship between HbA1c and plasmatic UA (32). Collectively, these results suggest that adequate circulating UA could protect against oxidative damage induced by high glucose. In this study, we compared the effects of the combined treatment of different doses of glucose and UA on lipid peroxidation.

Compared with the control and acute high-glucose treatment groups, the nucleus Nrf2 protein level was not significantly changed by UA supplementation at a dose of 300 μM, whereas it was suppressed in the high-glucose group, suggesting that Nrf2 signaling is associated with the favorable effect of adequate levels of UA. This speculation was supported by the observation of the upregulated *gpx7* and *gss* expression following UA supplementation at a dose of 300 μM. In line with this result, the physiological concentration of UA could provide antioxidative protection to cardiac myocytes treated with H_2_O_2_ by activating the Nrf2 pathway. UA could restore the hyperglycemia-induced down regulation of G protein (the α-subunit of inhibitory guanine nucleotide regulatory protein) by inhibiting the production of NO and the formation of ONOO^−^, which may have beneficial effects on improving the cardiovascular complications of diabetes (33). Hence, this result implies that adequate levels of UA may play a favorable role in partially alleviating the oxidative damage induced by acute exposure of high-level glucose by activating the Nrf2 pathway.

### A High Level of UA Increases the Oxidative Damage Induced by Acute Exposure of High Level of Glucose

A high concentration of UA can be a prooxidant, causing serial chain reactions of oxygen radicals and aggravating oxidative stress (25). As an important clinical manifestation, hyperuricemia is an important clinical symptom that is thought to be related to cardiovascular disease and occur secondary to a state of generalized vascular endothelial dysfunction, and it has been demonstrated to play a mechanistic role in cardiovascular disease by causing inflammation and generating oxidative stress in the vasculature (27). Epidemiological studies have reported that there is a close relationship between high levels of UA *in vivo* and some angiocardiopathy, nephropathy, and metabolic syndrome diseases (26). In our previous study, supraphysiological UA concentrations directly promoted oxidative damage in primary cultured chicken cardiac muscle cells and aggravated oxidative stress in H_2_O_2_-damaged cells (24). Hence, the role of high UA in high glucose condition was further investigated in this study.

In the presence of a high level of glucose (300 mM), treatment with a high UA concentration (1,200 μM) resulted in elevated levels of TBARS, ROS, and protein carbonyls, suppressed SOD activity, and reduced GSH concentration compared to treatments supplemented with UA (300 μM) or without UA, indicating increased oxidative damage in cardiac myocytes exposed to both high UA and high glucose. In accordance with this result, there is growing evidence to suggest that high circulating UA plays a causal role in the development of metabolic syndromes such as hyperglycemia, hypertriglyceridemia, and hypertension (28). In rats fed a high-fructose-enriched diet, the downregulated expression of antioxidant enzymes is linked to high plasma UA (30). In hyperuricemia men, serum UA was an explanatory variable for serum glycated albumin (31). The present study further demonstrated that high levels of UA exacerbated the oxidative damage that was induced by high glucose. In fructose-fed rats, the high blood UA level is restored to a normal level by resveratrol administration (66), suggesting that high blood glucose interferes with UA metabolism. Conversely, an increased UA level inhibits insulin receptor substrate 1 and Akt and induces insulin resistance via augmented ROS production and oxidative stress (67). Collectively, the result implies that high levels of UA may prevent insulin resistance by strengthening protection oxidative stress.

Compared to the high glucose treatment, nuclear Nrf2 was further decreased by combined glucose and UA treatment, in line with the downregulated *cat, sod1, sod2, gss*, and *gclc* gene expression. This result indicated that the combined treatment of high glucose and UA further suppressed the Nrf2 pathway, which is associated with enhanced oxidative stress. An attenuation of hepatic oxidative stress in fructose-fed rat livers after resveratrol administration was associated with increased nuclear levels of Nrf2 compared with other groups (66).

## Conclusion

Taken together, our systematic experiments prove that acute exposure of high levels of glucose induces oxidative damage in the cardiac myocytes of chicken. The present result suggests that an adequate level of uric acid is helpful in alleviating the oxidative damage that is induced by high glucose, whereas the blocked Nrf2 pathway by a high level of uric acid may render the cardiac myocytes more vulnerable to the effects of oxidative damage.

## Data Availability Statement

The raw data supporting the conclusions of this article will be made available by the authors, without undue reservation.

## Ethics Statement

The animal study was reviewed and approved by Guidelines for Experimental Animals established by the Ministry of Science and Technology.

## Author Contributions

XS and HL conceived and designed the experiments and wrote the paper. XS performed the experiments and analyzed the data. XW, JZ, and HJ provided essential reagents. All authors read and approved the final manuscript.

## Conflict of Interest

The authors declare that the research was conducted in the absence of any commercial or financial relationships that could be construed as a potential conflict of interest.
